# Intraspecific drought tolerance in Ugandan *Coffea canephora* for accelerated breeding selection

**DOI:** 10.1371/journal.pone.0349873

**Published:** 2026-05-26

**Authors:** Milton Ali, Settumba B Mukasa, Valerie Poncet, Godfrey Sseremba, Pierre Marraccini, Pascal Musoli, Daphne Nyachaki Bitalo, Hervé Etienne, Mildred Ochwo Ssemakula, Michael Kanaabi, Doreen Murenju Chelangat, Denis Fabre, Mildred Julian Nakanwagi, Sophie Léran, Anitah Tusiimire, Naome Aryatwijuka, Qurayish Musinguzi, Geofrey Arinaitwe, Boris Delahaie, Mathieu Gonin

**Affiliations:** 1 School of Agricultural Sciences, Makerere University, Kampala, Uganda; 2 National Coffee Research Institute, National Agricultural Research Organisation, Mukono, Uganda; 3 CIRAD, UMR DIADE, Montpellier, France; 4 UMR DIADE, Univ Montpellier, IRD, CIRAD, Montpellier, France; 5 National Crop Resource Research Institute, National Agricultural Research Organisation, Kampala, Uganda; 6 CIRAD, UMR AGAP Institut, Montpellier, France; 7 UMR AGAP Institut, Univ Montpellier, CIRAD, INRAE, Institut Agro, Montpellier, France; Swedish University of Agricultural Sciences, SWEDEN

## Abstract

Robusta coffee *(Coffea canephora*) accounts for about 80% of Uganda’s coffee production and supports over 2.2 million livelihoods. Yet recurrent droughts and erratic rainfall, exacerbated by climate change, pose severe threat to its productivity. Despite this threat, Ugandan breeding programs lack drought-tolerant varieties, largely because selection has not incorporated precise physiological traits linked to drought adaptation. Here, we evaluated 165 diverse Ugandan *C. canephora* genotypes, including local wild accessions, commercial lines, and breeders’ selections, under controlled screenhouse drought assay. Morphophysiological traits were collected before, during, and after drought stress. Using linear mixed-effects models, K-means clustering, and the Drought Factor Index, variation in drought tolerance was characterized across genotypes differing in biomass. Drought reduced water relations, gas exchange (gs and E), and PSII efficiency (Fv/Fm, Fo/Fv, ETR, Y(II), and PI) across all biomass groups. Nevertheless, 24 genotypes maintained higher DFI values, stable PSII function (Fv/Fm, PI, Fv/F₀), and less negative pre‑dawn water potential under severe stress. Among all traits, photosynthetic performance index (PI) and Fv/F₀ emerged as the most robust and biologically interpretable predictors of drought tolerance. These fluorescence‑based markers, together with the identified drought‑tolerant genotypes, provide a powerful foundation for accelerating climate‑smart coffee breeding in Uganda.

## Introduction

Rising global temperatures and erratic rainfall are intensifying across tropical agricultural systems, threatening the sustainability of crops critical to rural livelihoods [1,2]. Coffee, a cornerstone of tropical economies, is highly sensitive to these climatic disruptions [3–5]. Global production relies on *Coffea arabica* and *C. canephora* (Robusta), with the latter contributing ~40% of world supply and dominating Uganda’s coffee sector [6–9]. Despite its economic weight, Ugandan *C. canephora* yields an average of 500 kg/ha compared to 2500 kg/ha reached in other leading coffee-producing countries [10,11]. This yield gap stems largely from differences in production context, with high-yielding systems relying on optimized irrigation and over-fertilization [12,13]. In contrast, Ugandan smallholders operate under low-input conditions and face compounding biotic and abiotic stresses, including recurrent drought and erratic rainfall [14–18]. These interacting pressures disrupt water relations, photosynthesis, and reproductive development, thereby constraining yield realization [19].

Although *C. canephora is* often characterized as more heat- and disease-tolerant than *C. arabica*, this perceived hardiness collapses under prolonged water deficit [20–22]. Under drought, coffee deploys coordinated morphological and physiological adjustments to maintain water balance [4,23,24]. Plants reduce leaf area, adjust leaf inclination to limit solar interception, and develop deeper root architectures to scavenge soil moisture [25–27]. Physiologically, drought triggers rapid stomatal closure to conserve water [28–31]. However, this defensive strategy curtails CO₂ uptake, suppresses Rubisco activity, and promotes accumulation of reactive oxygen species (ROS) [19,28,32]. To avoid photo-oxidative damage, plants activate photoprotective pathways, primarily non-photochemical quenching (NPQ), which shows as declines in maximum PSII quantum yield (Fᵥ/Fₘ), operational efficiency (ΦPSII) and increased thermal dissipation [33–35].

Despite these well-documented mechanisms, drought-response dynamics remain poorly resolved in Uganda’s diverse *C. canephora* germplasm [36,37]. Previous studies have relied mainly on phenotyping feral, wild, and half-sib accessions using terminal growth traits [38,39]. Because these endpoint phenotypes integrate cumulative stress damage rather than underlying physiological processes, they exhibit low heritability, high environmental noise, and cannot identify failure thresholds or adaptive capacity [40,41]. As such, key functional traits with direct mechanistic relevance to drought tolerance, including predawn leaf water potential, gas exchange kinetics, and dynamic chlorophyll fluorescence, remain excluded from selection pipelines. This omission forces breeders to rely on post-hoc survival proxies, slowing breeding cycles and genetic gain for complex polygenic traits like drought tolerance.

To overcome these bottlenecks, crop improvement is shifting toward integrative phenotyping that links physiological function to stress tolerance [42,43]. Multivariate approaches such as principal component analysis (PCA), hierarchical clustering, and linear discriminant analysis have been widely used to differentiate drought-tolerant genotypes [42–45]. However, these methods often treat trait covariation as statistical patterns rather than physiological drivers, limiting their predictive power. The Drought Factor Index (DFI) offers a more direct approach by quantifying the extent and intensity of abiotic stress effects on photosynthetic performance, providing a physiologically relevant marker of genotype performance [46–49]. While DFI has proven useful in selecting drought- and salt-tolerant genotypes in barley, tobacco, and sorghum, its applicability for perennial crops like coffee remains untested [46,47,49]. By capitalizing on Uganda’s extensive *C. canephora* diversity, this study aims to 1) describe drought tolerance response variation among a large set of Ugandan *C. canephora* accessions, 2) identify drought-resilient *C. canephora* genotypes by using DFI alongside K-means classification, and 3) assess the morphophysiological response of drought-tolerant and sensitive genotypes.

## Materials and methods

### Permits and ethical considerations

No specific permits or licenses were required for this study. All plant materials used are conserved within the *ex situ* germplasm collections of the National Coffee Research Institute (NaCORI), Kituza Station, under the National Agricultural Research Organization (NARO), Uganda, and are routinely used for research and breeding activities. The work did not involve endangered or protected species, nor did it require access to privately owned land or engagement with human participants. As such, no additional field access approvals or consent was necessary.

### Plant material

A total of 165 Ugandan *C. canephora* genotypes were evaluated, representing four categories according to their cultivation status: 123 breeder selections collected from farmers’ fields across major Robusta-growing regions, 19 advanced breeders’ lines derived from controlled crosses, 23 wild accessions sampled from Zoka, Budongo, and Marabigambo forests; and 10 National Agricultural Research Organisation (NARO) commercial varieties referred to as KR clones. The genotypes were selected from all sources to capture key traits beneficial to Robusta variety improvement in Uganda, including resistance to coffee wilt disease, high yield potential, large bean size, desirable cup quality, wide genetic and phenotypic diversity [15,16,36,50–52].

### Clonal propagation

Genotypes were regenerated in 2023 by cloning via macro-stem cuttings obtained from mother gardens maintained at the NaCORI ex-situ collection found at Kituza station in Mukono district of Uganda. Healthy suckers were harvested and trimmed into single-node cuttings, which were then dipped in rooting hormone powder (Seradix 2, 0.8% w/w indole-3-butyric acid, Twiga Chemicals Industries, Nairobi, Kenya). The cuttings were planted in a seedbed containing a rooting horticultural substrate composed of topsoil and sand mixed in a ratio of 3:1 by volume and covered with an 800-micron white transparent polyethylene sheet to reduce evapotranspiration. After nine months, rooted plantlets of uniform size were transplanted into 5 x 7 cm poly pots filled with an establishment substrate mixture of black soil, lake sand, and composted manure in a ratio of 3:2:2 by volume. The young plants were regularly watered and acclimatized gradually to screenhouse conditions by incrementally removing the polyethylene covers to decrease relative humidity and increase light.

### Experimental design and drought stress application

**Fig 1:**
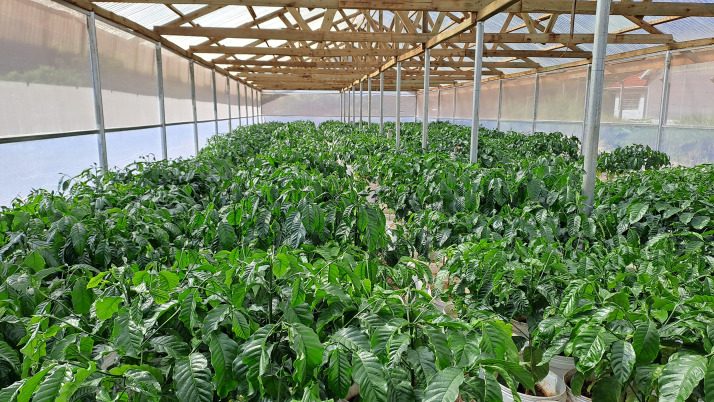
One year and seven-month-old *C. canephora* plantlets grown in 20-liter plastic buckets within a screenhouse roofed with a white transparent roof, fine mesh netting for ventilation, and white polyethylene sheet walls were used for drought stress evaluation under controlled conditions at NaCORI.

The drought assay was conducted from 7^th^ July to 15^th^ November 2024 in a controlled screenhouse, measuring 8 by 24 meters, set up with a transparent roof (>90% light transmittance), fine mesh netting on the upper sides for ventilation, and white polyethylene sheet walls to prevent rainwater intrusion (Fig 1). Three plants per genotype, all 11 months old at the time of transplanting and with a single stem, were arranged randomly in a row and column design. Each plant was placed in a 20-liter perforated plastic bucket filled with 1 kg of stone aggregates for drainage, topped with a mixture of loam soil, cow dung manure, and sand in a 3:2:2 (v/v) ratio. Plants were irrigated with 700 ml every two days during the first five months, then increased to 1500 ml every two days for 2 months to meet the water needs of bigger plants. Morphophysiological measures in sequential batches of 16 genotypes throughout the experimental period were taken within one day before withholding water to serve as a well-watered control (WW). Genotypes were then withheld water for 15 days to induce drought stress (DS) throughout the experiment. The drought stress period was monitored through water potential measurements and finalized when most plants reached a reading of −3.5 MPa.

### Data collection

#### Temperature and relative humidity monitoring.

Microclimatic conditions within the screenhouse were monitored daily using seven dual-channel temperature and humidity data loggers (Tinytag Plus 2, TGP-4500; Gemini Data Loggers Ltd., UK) installed across the screenhouse to have a good spatial distribution of Tinytag sensors. Hourly recordings were taken throughout the experimental period. Prior to the onset of drought stress (May to mid-July 2024), the maximum daily temperature ranged from 28°C to 32°C, with peaks observed in July, while minimum temperatures varied between 20°C and 24°C (*S1 Fig*). Relative humidity remained high during this period, with daily maximums ranging from 75% to 90% RH and minimums between 60% and 70% RH. During the drought phase (July to November 2024), the screenhouse environment exhibited relatively stable conditions, with maximum temperatures maintained between 30°C and 32°C and minimum temperatures between 22°C and 24°C. Relative humidity also remained the same as in the pre-stress period.

### Phenotyping of coffee growth and morpho-physiological parameters

Coffee growth and morpho-physiological traits were assessed in 18-month-old plants. Screening was conducted in batches, each lasting for 15 days. For each batch, measurements were taken at three time points: (i) baseline (immediately before stress imposition); (ii) daily during the drought phase to track stress progression; and (iii) at the termination phase on day 15 to capture drought  cumulative drought effects (Table 1). These points were selected to characterize genotype responses across the drought assay, and data were collected throughout the experiment period to ensure consistency. The entire screening spanned from 15^th^ July to 15^th^ November 2024.

**Table 1:** Traits measured during the different phases of drought stress in C. canephora.

Phase	Traits	Units	Physiological relevance
Before Drought	Plant height	Cm	Indicate overall vertical growth
	Stem internodes	Count	Indicates plant elongation
	Canopy height/diameter	Cm	Vertical and lateral growth
	Number of primaries/internodes	Count	Reflects branch development and elongation
	Length of the longest primary	Cm	Indicates horizontal canopy growth
	Stem girth	Mm	Reflects plant vigor
	Number of leaves	Count	Indicates overall vegetative growth
	Leaf length/width	Cm	Reflects leaf size and growth potential
	Leaf Surface Area	cm²	Influences transpiration and photosynthesis capacity
	Total Leaf Area	cm²	Reflect overall plant biomass
	Leaf inclination (Leafincl)	score (1–3)	Protective mechanisms against water stress
	Pre-dawn leaf water potential (PDLWP)	Mpa	Indicates water status and soil matrix potential
	Stomatal conductance (gs)	mol H₂O m ⁻ ² s ⁻ ¹	Regulates gas exchange, an early stress signal
	Fv/Fm	–	Measures efficiency of photosystem II (PSII)
	Fv/Fo	–	Measures photochemical activity of PSII
	Fo/Fm	–	Measures the non-photochemical quenching of PSII
	YII	–	Measures actual efficiency of PSII electron transport
	ETR	µmol e^-^ m ⁻ ² s ⁻ ¹	Measures active photosynthetic electron flow
	Transpiration rate (E)	E-mmol H₂O m ⁻ ² s ⁻ ¹	Indicates total water loss; reduced under drought
	Chlorophyll content (Chl)	–	Proxy for photosynthesis; decreases under stress
	Performance Index (PI)	–	Composite PSII performance indicator
Drought phase	Leaf Leafincl, PDLWP, gs, E, ETR	–	Same as role as before drought stress
	Chl, Fv/Fm, Fv/Fo, Fo/Fm, YII, PI	–	Same as the role as before drought stress.
Termination of drought phase	Coffee growth parameters	–	Same role as before, drought stress.
	Leafincl, PDLWP, gs, E, ETR,	–	Same role as before, drought stress.
	YII, Chlo, Fv/Fo, Fo/Fm, Fv/Fm, PI	–	Same role as before, drought stress.

### Trait Measurements

Measurements of morphological and physiological variables were taken between 08:30 and 11:30 a.m. to minimize diurnal variability across batches using fully expanded leaves on the third node from the tip of the third primary branch for consistency [53,54]. Trait selection was informed by previous studies that demonstrated their relevance as indicators of plant responses to water deficit and as proxies for drought tolerance [34,35,55].

### Pre-dawn leaf water potential

Pre-dawn leaf water potential (Ψ _PDLWP_) was measured using a Scholander pressure chamber (Model 600, PMS Instrument Company, USA) at baseline, upon initial signs of leaf inclination, and subsequently at every two-day interval until Ψ_PDLWP_ declined to around −3.5 MPa, corresponding to high drought stress for coffee plants grown under controlled conditions [56]. Target leaves were enclosed in aluminum pockets for one hour to block light, close stomata, and allow equilibration of leaf water potential with soil moisture to mimic the pre-dawn conditions [57]. The petiole was excised with a surgical blade and inserted into the chamber. Compressed gas was gradually applied, and the pressure at which xylem sap appeared was recorded as Ψ _PDLWP_.

### Photosynthetic and chlorophyll fluorescence parameters

Photosynthetic parameters, including stomatal conductance (gs), transpiration rate (E), electron transport rate (ETR), and effective quantum yield of PSII (Y(II)), were measured using a MINI-PAM-II/ B photosynthesis analyzer with a porometer (Heinz Walz GmbH, Germany) under ambient light [58]. Chlorophyll fluorescence parameters, including maximum quantum yield (Fv/Fm), variable to minimal fluorescence ratio (Fv/Fo), minimal to maximal fluorescence ratio (Fo/Fm), and performance index (PI), were determined using a Pocket PEA fluorimeter (Hansatech Instruments Ltd., UK) after dark-adapting the leaves for 40 minutes with light-exclusion clips to ensure complete stabilization of photosystem II (PSII) [59].

### Chlorophyll content

Relative chlorophyll content was estimated non-destructively using a SPAD-502 Plus chlorophyll meter (Konica Minolta, Japan), which calculates values based on light transmittance at 650 nm and 940 nm. SPAD readings from three leaves per plant were averaged and recorded as a single value in the field book app [60].

### Plant growth and morphological traits

**Fig 2:**
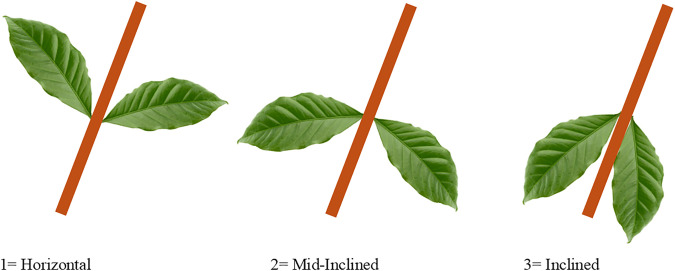
Leaf inclination scale showing drought stress progression in *C. canephora*  where 1 = horizontal, fully turgid; 2 = mid-inclined, early sagging; 3 = inclined, pronounced downward sagging.

Leaf inclination was recorded daily using a standardized three-point visual scale (Fig 2).

Plant height was measured from the substrate surface to the apical meristem using a builder’s tape measure (±0.01 cm). Canopy height was recorded as the vertical distance from the first node to the apex, and canopy diameter was measured as the maximum horizontal spread of the branches. The length of the longest primary branch was measured from its point of attachment on the main stem to the distal tip. Leaf dimensions were taken from three fully expanded leaves per plant (typically from the third node from the apex). The leaf surface area was determined using the LeafArea mobile application (v 2.4; Android/iOS). The total leaf area per plant was computed by multiplying the leaf surface area by the total number of leaves. Stem girth was measured 3 cm above the soil surface using a digital caliper (±0.01 mm). Count-based architectural traits, including the number of stem internodes, primary branches, and fully expanded leaves, were recorded for each plant. All data were collected on an individual-plant basis and digitally captured in the field book [60].

### Data analysis

All statistical analyses were conducted using R version 4.4.3 [61].

### Morphological variation analysis among the *C. canephora* accessions

To account for inherent variation in plant size and vigor that could confound stress response assessments, *C. canephora* accessions were clustered using twelve non-destructive vegetative growth parameters (Table 1) as composite indicators of aboveground biomass. These traits were averaged at the genotype level and used to group accessions into phenotypically homogeneous classes via K-means clustering, with the optimal number of clusters determined by the elbow method (within-cluster sum of squares) [62]. To validate this partitioning, principal component analysis (PCA) was performed on the scaled genotype-level growth data using FactoMineR and factoextra [63]. The resulting cluster membership was subsequently used to define phenotypic biomass groups for downstream analyses. This approach allowed stress-response variation to be evaluated independently of pre-existing variation in growth stature.

### Comparative analysis of morphophysiological traits under increasing drought stress

Morphophysiological traits were analyzed to assess trait variation across the experiment using linear mixed effects models fitted separately for each trait across repeated time measurements. The general model structure was:


$$\begin{array}{c} Y_{jklm}=\mu +{f(D}_{j})+f(D{j)C}_{k}+C_{k}+Bm+G_{l}+P(l)+\varepsilon_{jklm} \end{array}$$

Where Y_jkℓm_ is the observed morphophysiological response variable for the ℓᵗʰ genotype at day *j*, belonging to biomass cluster *k* and batch *m*, μ is the overall mean; ${f(D}_{j})$ represents the effect of time modeled as a continuous variable using natural spline functions (ns, df = 2); ${f(D}_{j})\;x\;Ck$ *is the interaction between time and cluster; C*_*k*_ *is the fixed effect of the k^th^ cluster (biomass group); B*_*m*_ *is the fixed effect of batch; G*_*l*_ *is the random effect of the ℓ^th^ genotype; P*(l) is the random effect of plant nested within genotype; and ε_jkℓm_ is the residual error term, with ε_jkℓm_∼N(0,σe2).

Random effects were included to account for repeated measurements and genetic structure. The models were fitted by restricted maximum likelihood (REML) with fixed-effect significance assessed using Type III F-tests on the full mixed-effects models, with Satterthwaite’s approximations for degrees of freedom implemented in the lmerTest [64,65].

Leaf inclination (Leafincl), treated as an ordinal variable, was analyzed using cumulative link mixed models implemented in the ordinal package. The same fixed and random effects structure was applied, and the significance of time, cluster, and their interaction was assessed using likelihood ratio tests (LRT) comparing nested models.

Assumptions of normality and homogeneity of variance were verified through inspection of residual diagnostics. Group differences were visualized using trend plots based on model-derived estimated marginal means, as well as supplementary graphical representations (violin plots, bar plots) generated using ggplot2 [66,67].

### Drought Factor Index (DFI) calculation and trait-based analysis of tolerance

Genotypes were ranked for drought tolerance (D^T^), intermediate (D^I^) and sensitivity (D^S^) using the DFI [49]. Photosynthetic performance index measurements were considered at three key time points: pre-stress (control, day 1), one week of water stress (moderate, day 7), and two weeks of water stress (severe, day 15). Genotype-specific values were obtained from a mixed model implemented in the lme4 package. The model included batch and day (treated as a categorical factor) as a fixed effect, while genotype-specific deviations across time were modeled as random effects. Repeated measurements within plants were accounted for using a nested random effect structure with plant nested with genotype. From this model, best linear unbiased predictors (BLUPs) were extracted for each genotype at each time point. Relative performance under stress was calculated by normalizing genotype-specific PI values at stress conditions to the corresponding control baseline value (day 1), as shown in the formulas [49]:


$$\begin{array}{c} Relative\_PI\_Moderate=\frac{Moderate\;Stress}{Control\_PI}\;and\;Relative\_PI\_Severe=\frac{Severe\;Stress}{Control\_PI} \end{array}$$

The DFI was computed as a weighted log-transformed function of these relative values following the formula [49]:


$$\begin{array}{c} DFI=log\;(Relative\;PI\_Moderate)+\text{2}\;X\;log(Relative\;PI\_Severe) \end{array}$$

Genotypes were subsequently classified into drought response groups using k-means clustering applied to the DFI values. The optimal number of clusters was determined using the elbow method based on within-cluster sum of squares, resulting in three categories being identified: tolerant, intermediate, and sensitive.

### Correlation and regression analyses of DFI determinants

To identify morphophysiological traits that best predict drought response, we evaluated genotype-level associations among traits and their relationship with the drought factor index (DFI). Genotype morphophysiological trait values on day 1 (WW conditions) and day 15 (DS conditions) were derived as estimated marginal means from the linear mixed model described above, with batch included as a fixed effect. Pairwise trait correlations were then computed, and relationships among traits under WW and DS conditions were evaluated using Pearson correlation and regression analyses [68]. To identify baseline traits predictive of drought response, linear regressions were fitted using DFI as the response variable and each WW (day 1) and DS (day 15) derived adjusted trait (estimated marginal means) as an explanatory variable [69]. Model fit and relative predictive performance were quantified using adjusted R² values.

### Comparison between drought tolerant and sensitive genotypes

To compare physiological responses between drought-tolerant and drought-sensitive genotypes, as identified by k-means clustering, morphophysiological traits were evaluated at two key stages of the experiment corresponding to the onset of stress (Day 1, WW conditions) and the end of the stress period (Day 15). For each trait, linear mixed models were fitted to the subset of data corresponding to these time points, including batch and day as fixed effects and their interaction, with genotype included as a random effect. Estimated marginal means were extracted from the fitted models to compare D^T^ and D^S^ groups and are presented with their 95% confidence intervals.

## Results

### Biomass-based stratification of *C. canephora* accessions

To minimize size-related confounding effects, plants were first stratified to ensure accurate comparisons of trait responses among genotypes with similar water demand based on K-means clustering. The elbow criterion (k = 2) and PCA confirmed two distinct groups based on biomass: cluster 1 of small biomass (blue) and cluster 2 of large biomass (red) (S2 Fig). Cluster separations were primarily driven by biomass-related traits along PC1 (39.7%) of variance, while PC2 (17.5%) captured variation in leaf area. The large-biomass cluster contained 96 individuals, while the small-biomass cluster included 69 individuals. Both clusters contained accessions from all cultivation origins, indicating that biomass divergence was independent of cultivation history.

### Overview of drought-induced trait changes from the onset to the end of stress

Drought stress (DS) affected all measured traits (S1 Table). Stomatal conductance (gs) and transpiration rate (E) under DS exhibited the highest variability, as indicated by their coefficients of variation (CV).

**Fig 3:**
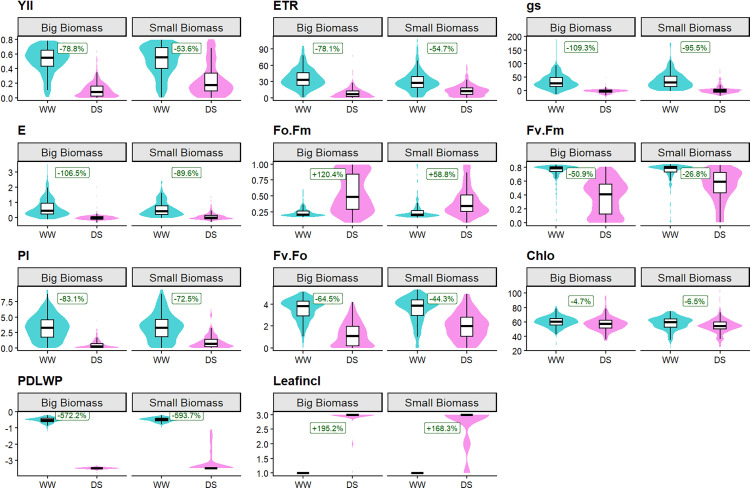
Violin plots showing morphophysiological trait responses of *C. canephora* biomass clusters between the onset and the end of drought stress. Each panel compares trait distributions at the onset of stress (Day 1, light blue) and at the end of the stress period (Day 15, pink) for the small (left) and large (right) biomass clusters. Boxes represent the median and interquartile range, and shaded violins show the distribution of observations. Percentage values indicate the relative change between Day 1 and Day 15, calculated from cluster-level median values. Traits displayed included (1) Fo.Fm, (2) Chlo, (3) Fv.Fm, (4) Fv.Fo, (5) PI (6) Y(II), (7) gs, (8) E,(9) ETR, (10) PDLWP, and (11) LeafIncl.

Based on percentage changes between the onset and the end of the stress period (Fig 3), genotypes in the large-biomass cluster showed greater reductions than in the small-biomass cluster: PI declined by 83.1% vs 72.5%, ETR by 78.1% vs 54.7%, Fv/Fm by 50.9% vs 26.8%, and YII by 78.8% vs 53.6%. Similarly, gs declined by 109.3% vs 95.5%, and E decreased by 106.5% vs 89.6%. PDLWP declined strongly by 572.2.0% and 593.7%.

In contrast, Chlo remained relatively stable (<10% reduction) across the biomass clusters. FO/FM rose by 120.4% vs 58.8%, leaf inclination (LeafIncl), treated as an ordinal trait, showed a clear shift in both clusters, with values moving from predominantly non-inclined (score 1) to fully inclined leaves (score 3).

### Temporal dynamics of morphophysiological trait responses to drought stress

Drought stress affected all measured physiological traits across genotypes over time (S3 Fig). Spline-based linear mixed models revealed strong effects of time and significant time x cluster interactions for most traits, indicating that differences between biomass groups emerged primarily over the course of the stress period (Table 2). Models also included batch as a fixed effect and genotype and plant identity as random effects (see Methods); however, for clarity, only effects relevant to the biological hypotheses (time, cluster, and their interaction) are presented here, while full model outputs are provided in (SI Table 2).

**Table 2:** Mean square values for morphophysiological traits in C. canephora clusters under drought stress and well-watered conditions across all measurement points.

Trait	Day	Biomass Cluster	Day: Biomass Cluster
Chlo	2136.47***	250.29**	198.91**
E	65.56***	0.2ns	1.00***
ETR	84670.03***	2668.03***	5575.26***
Fo/Fm	10.00***	0.05ns	1.35***
Fv/Fm	13.73***	0.06ns	1.94***
Fv/Fo	569.04***	1.31ns	38.47***
PDLWP	101.24***	24.65***	3.28***
PI	1089.21***	0.16ns	19.58***
YII	16.94***	0.01ns	0.52***
gs	233400.38ns	1125.23**	351.98ns
Leafincl	***	***	***

*** significant level p ≤ 0.001, ** significant level p ≤ 0.01, * significant level p ≤ 0.05, ns, not significant.

At the onset of stress (Day 1), all genotypes exhibited very limited variation for PDLWP and leafincl, whereas greater variability across genotypes was observed for ETR, gs, E, PI, YII, Fo/Fm, Fv/Fm, and Fv/Fo. From approximately Day 5 onward during the stress period, distinct patterns associated with biomass became apparent. Genotypes in the small biomass cluster maintained higher and more gradually declining values of PDLWP, gs, E, and photochemical traits (Fv/Fo, Fv/Fm, YII, PI), rather than complete stability. In contrast, the large biomass cluster displayed steeper and earlier declines in ETR, PI, and YII, accompanied by an increase in Fo/Fm as early as Days 5–7, together with marked reductions in PDLWP, gs, and E, reflecting higher vulnerability to water limitation.

Notably, although mean trajectories differed clearly between clusters (Table 2, S3 Fig), genotypes within the same biomass group still exhibited heterogeneous physiological responses, indicating that biomass-based stratification does not fully capture the diversity of drought response.

### Selection of drought-tolerant genotypes across cultivation status and clusters

DFI values varied widely among genotypes (−4.93 to +2.40), indicating substantial variation in drought response. Based on k-means clustering, genotypes were classified into three groups representing 24 tolerant (D^T^), 91 intermediate (DI), and 50 sensitive (D^S^) genotypes (S4 Fig).

**Fig 4:**
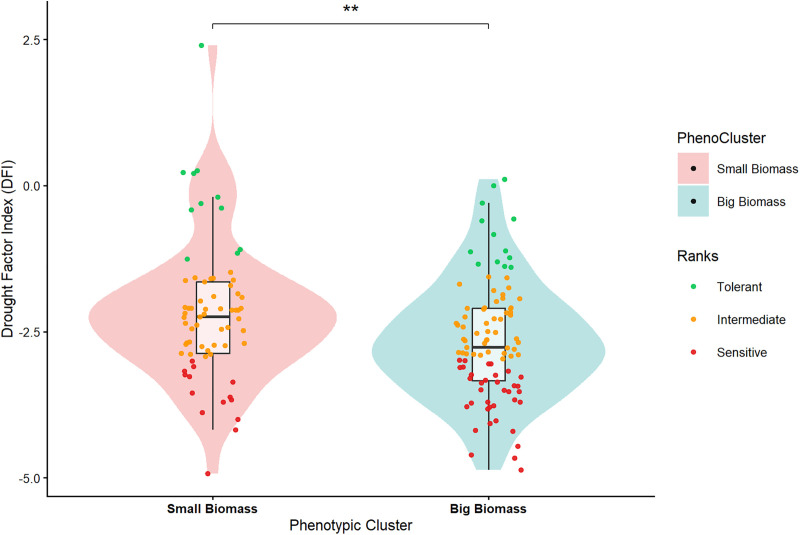
Distribution of drought-tolerant and sensitive genotypes across the biomass clusters. Violin plots showing the Drought Factor Index (DFI) across two phenotypic clusters under drought stress. Each dot represents a genotype (green = tolerant, orange = intermediate, and red = sensitive). Boxes indicate interquartile ranges with median lines, **p ≤ 0.001) significant difference between clusters; overlap of red and green dots within clusters reflects genotype-specific variation in drought response.

The tolerant group was characterized by higher DFI values, reflecting greater stability of photosynthetic performance under drought conditions, whereas sensitive genotypes showed strongly negative DFI values. The slightly positive DFI observed for 5 genotypes (Table 3) reflects a relative stability of photosynthetic performance under drought rather than an absolute increase in PI (Fig 4).

**Table 3:** Top 24 drought-tolerant C. canephora genotypes ranked by DFI.

Genotype	PhenoCluster	Cultivation status	DFI
288/11	Small Biomass	Breeders selection	2.40
266S/25/1	Small Biomass	Breeders selection	0.26
ZKO	Small Biomass	Zoka forest	0.23
266S/11/3	Small Biomass	Breeders selection	0.21
226/65/1	Big Biomass	Breeders selection	0.11
288/12	Big Biomass	Breeders selection	0.00
KR1	Small Biomass	KR line	−0.19
1/30//2	Big Biomass	Breeders line	−0.30
BDN S5	Small Biomass	Budongo forest	−0.31
261S/15/1	Small Biomass	Breeders selection	−0.38
EU /17	Small Biomass	Breeders selection	−0.41
Q/1/1	Big Biomass	Breeders selection	−0.57
UFCT3	Big Biomass	Breeders selection	−0.60
209/29/8	Big Biomass	Breeders selection	−0.84
234/37/5	Small Biomass	Breeders selection	−1.09
254/28/4	Big Biomass	Breeders selection	−1.12
J24/13/59/4	Big Biomass	Breeders selection	−1.13
Kanengo 2/15	Small Biomass	Breeders selection	−1.15
BDN S13	Big Biomass	Budongo forest	−1.23
BD L8 T13	Small Biomass	Budongo forest	−1.26
266S/11/6	Big Biomass	Breeders selection	−1.30
BD L8 T6	Big Biomass	Budongo forest	−1.34
KR2	Big Biomass	KR line	−1.38
EU /3	Big Biomass	Breeders selection	−1.40

Across clusters, all D^T,^ DI, and D^S^ genotypes were identified. The small biomass cluster contained 11 D^T^ genotypes (green dots), 44 D^I^ (orange dots), and 14 D^S^ (red dots) (Fig 4), whereas the big biomass cluster had 13 D^T^ genotypes, 47 DI, and 36 D^S^ genotypes. The D^T^ genotypes originated from all the cultivation statuses (S1 Table). The distribution of D^T^ and D^S^ genotypes differed significantly between biomass clusters.

### Trait interrelationships and predictors of drought tolerance

To identify morphophysiological traits capable of predicting drought resilience under baseline (Day 1) and at the termination of drought (Day 15), we conducted a Pearson correlation analysis. Under WW conditions, chlorophyll fluorescence parameters exhibited a strong correlation with DFI, with Fv/F₀ (r = −0.75), PI (r = −0.74), and Fv/Fm (r = −0.61) showing significant negative associations. In contrast, gas exchange traits (gs, E, and ETR), water status (PDLWP), Chlo, and LeafIncl exhibited negligible associations with DFI (r < −0.22). To determine whether trait DFI relationships persist once drought is established (day 15), we repeated correlation, and no trait maintained a significant correlation with DFI (r < 0.26) (S5 Fig).

Linear regression analyses confirmed the predictive utility of baseline fluorescence traits. PI and Fv/Fo explained 55% and 57% of the variance in the DFI, respectively (R² adj. 0.55 and 0.57) (S6 Fig). While gas exchange parameters (ETR, gs, E), water status (PDLWP), and Chlo also exhibited negligible predictive power at baseline (R²adj < 0.05). Once drought was established by day 15, all predictive signals collapsed (S7 Fig).

### Morphophysiological response of drought-tolerant and sensitive genotypes to drought stress

Based on the k-means classification of genotypes according to their drought response (DFI, see above), drought-tolerant and drought-sensitive groups were compared for morphophysiological traits at two key time points: the onset (Day 1) and end of stress (Day 15) (Fig 6).

Under WW conditions, D^S^ genotypes consistently exhibited higher values for PI, YII, Fv/Fm, gs, and ETR compared to D^T^ genotypes, indicating higher baseline physiological activity. In contrast, under D^S^ conditions (Fig 6), D^T^ genotypes maintained significantly higher gs, ETR, PI, Fv/Fm, and YII, suggesting their ability to sustain photosynthetic performance under stress. Overall, these results highlight a shift in relative performance between groups, with sensitive genotypes exhibiting higher initial values under WW conditions, but stronger declines under drought stress compared to tolerant genotypes.

## Discussion

Drought stress is a serious abiotic constraint to *C. canephora* production, affecting photosynthesis, disrupting key metabolic processes, and curtailing physiological performance [4,35,70,71]. In this study, imposing drought stress significantly lowered physiological performance across *C. canephora* genotypes, irrespective of their biomass clusters (Table 2, Fig 3, S3 Fig). Although both clusters showed declines relative to the WW baseline, the magnitude of reduction varied sharply between them (S3 Fig). Genotypes in the big-biomass cluster experienced greater declines in PDLWP, Leafincl, gs, E, ETR, and in photosynthetic efficiency parameters (PI, YII, Fv/Fm) (Fig 3). These reductions point to impaired hydraulic control, excessive transpiration, and restricted capacity for carbon assimilation under stress [72–74]. In contrast, small-biomass genotypes displayed a more moderate decline in these parameters, consistent with lower water use and conservative transpiration [27,72,75,76]. This divergence between the clusters suggests that tolerance is genotype-specific rather than solely determined by plant size. Similar trade-offs were reported in wheat and maize, where compact architectures are associated with cooler canopies, delayed senescence, and heightened drought tolerance [77–79]. These observations suggest that breeding for drought tolerance in *C. canephora* should prioritize specific physiological resilience traits rather than biomass or vigor alone, as tolerance can occur independently of plant size [74,80].

**Fig 5:**
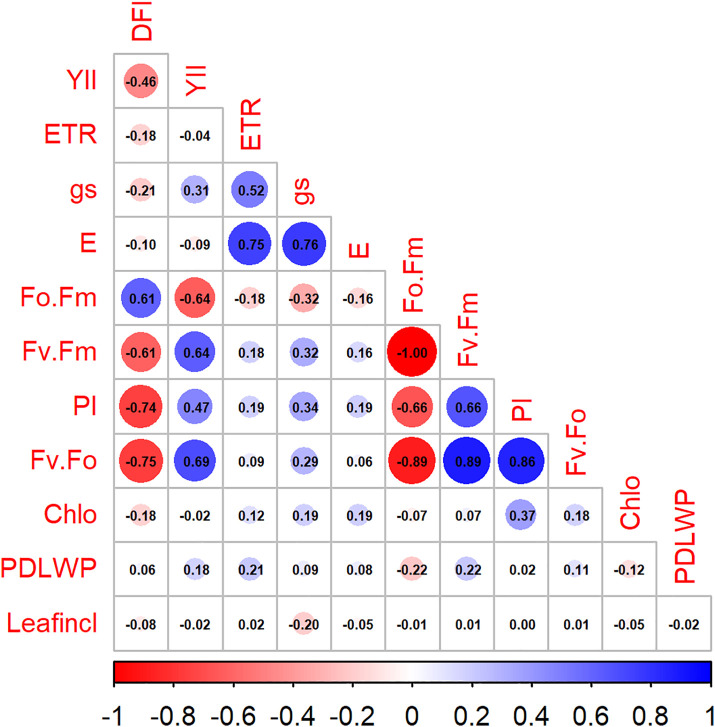
Pearson correlations among morphophysiological traits in *C. canephora* on day 1 of WW condition. The heatmap displays correlation. among the DFI, Y(II), ETR, Fv/Fm, Fv/Fo, Fo/Fm, PI gs, E Chlo, PDLWP, and Leafincl. Circle color denotes the direction of correlation (blue = positive; red = negative), while circle size reflects the strength of the association (larger circles = stronger correlations).

Correlation and regression analyses revealed that baseline fluorescence traits emerged as the strongest early predictors of drought resilience in *C. canephora*. Under WW conditions (Day 1), Fv/F₀, PI, and Fv/Fm showed strong negative correlations with DFI (r = –0.75, –0.74, and –0.61; Fig 5). These signals translated into substantial predictive power, with PI and Fv/F₀ explaining 55% and 57% of DFI variance. Gas‑exchange traits (gs, E, and ETR), water‑status (PDLWP), chlorophyll content (Chlo), and leaf inclination (LeafIncl) showed negligible associations (r < −0.22; R²adj < 0.05) (S6 Fig). Once drought was established, all trait–DFI relationships collapsed (r < 0.26; S5 Fig and S7 Fig), indicating that severe stress drives physiological convergence and masks genotype-level differences [81]. This pattern aligns with reports that early photochemical efficiency is more heritable and genotype‑specific than late‑stage stress responses, which often saturate across genotypes [82,83]. The strong performance of PI and Fv/F₀ is consistent with their sensitivity to PSII damage and energy‑dissipation dynamics, which are tightly linked to drought tolerance across perennial crops [84]. Together, these results position baseline PI and Fv/F₀ as robust, scalable, and biologically interpretable indicators for early selection of drought‑resilient coffee genotypes.

Improving drought tolerance in *C. canephora* remains a challenge because the trait is complex and polygenic in nature [85,86]. Traditional multivariate techniques such as hierarchical clustering, PCA, and linear discriminant analysis have been used to single out genotypes that can withstand drought stress [42–44]. However, they fail when trying to capture the ever‑shifting web of trait interactions while ranking genotypes based on functional drought resilience [42–44]. The drought factor index (DFI), which takes into consideration the intensity and duration of stress across a suite of physiological signals, overcomes these hurdles by linking adaptive performance squarely to photosynthetic performance [47–49,87]. While previously applied to cereals such as barley and sorghum [46,49], this study presents its first comprehensive application in *C. canephora*. Using DFI, 165 genotypes were evaluated, and 24 high performant accessions were identified as drought tolerant (Table 3, *S4 Fig* and Fig 4). These tolerant genotypes came from both biomass clusters and across cultivation statuses (breeder selections, breeders’ lines, KR commercial lines, and natural forest populations), suggesting that drought tolerance is not linked to plant size or cultivation status. This finding contrasts with earlier reports suggesting a close association between vigor and drought response in coffee [80,88]. The broad distribution of tolerant genotypes across cultivation status points to genetic variation as the primary driver of drought resilience in *C. canephora* rather than the growth habit or cultivation status [38,88].

**Fig 6:**
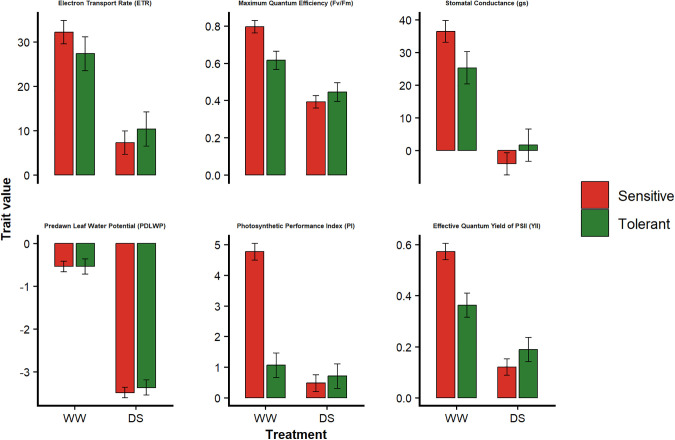
Physiological responses of drought-tolerant (D^T^) and drought-sensitive (DS) *C. canephora* genotypes at the onset of stress (no stress, day 1) and at the end of stress (day 15). Bars represent model-derived estimated marginal means (± 95% confidence intervals) for ETR, Fv/Fm, gs, PDLWP, PI, and YII. Genotypes were classified into tolerance groups based on k-means clustering of drought response indices.

Under WW conditions, sensitive genotypes showed significantly higher chlorophyll fluorescence (Fv/Fm, Fv/Fo, and Y(II)) than tolerant ones (Fig 6). This suggests a vigorous photosynthetic strategy that maximizes carbon gain and depends heavily on sufficient water supply [89,90]. Such high fluorescence under non-stressed conditions, was also reported in other crops, and often signifies fast-growing genotypes with limited regulatory capacity under water deficit [89,91]. In contrast, tolerant genotypes maintained higher Fv/Fm, Fv/Fo, PI, gs, ETR, and less negative PDLWP under drought stress, depicting enhanced photochemical stability, regulated stomatal control, and effective water conservation, key attributes of drought adaptation [92,93]. These patterns indicate that drought tolerance in *C. canephora* depends on sustaining leaf water potential and coordinated control of photosynthetic and stomatal functions. Such traits represent reliable physiological indicators and breeding targets for improving coffee drought resilience.

Overall, this study demonstrates that drought tolerance in *C. canephora* is physiologically anchored in the coordinated maintenance of leaf water potential, photosynthetic stability, independent of plant size or cultivation status. The identification of tolerant genotypes across biomass clusters and cultivation status underscores the complexity and polygenic nature of drought resilience in coffee.

Although this study identified drought‑tolerant varieties, extrapolation of these findings to field conditions must be made with caution. Container‑grown plants experience constrained rooting volumes that can modify root system architecture, hydraulic conductance, and soil–plant signaling, often accelerating soil drying and intensifying drought stress relative to open‑field soils [94,95]. Such systems also limit access to deep or heterogeneous soil profiles and buffer plants from the spatial and temporal variability in microclimate, soil moisture, and biotic interactions that shape drought adaptation under natural environments [96,97]. As such, trait expression, particularly for parameters linked to water uptake, stomatal regulation, and canopy transpiration, may differ in magnitude or stability compared with field‑grown plants [98]. While the controlled screenhouse enabled standardized stress imposition and precise physiological phenotyping, several experimental limitations persist. The relatively short‑lived drought period restricts assessment of chronic stress responses and post‑stress recovery dynamics. In addition, it cannot fully capture long‑term acclimation processes or genotype × environment interactions documented in field studies [94,95]. Field validation across diverse agroecological settings will therefore be essential to confirm the agronomic relevance, yield stability, and durability of the drought‑tolerant genotypes identified here.

Future studies should focus on dissecting the anatomical, biochemical, and molecular mechanisms underlying these physiological traits, integrating multi-omics analyses to unravel the regulatory networks that confer stability under water deficit. Beyond physiological markers, linking drought tolerance with agronomic performance, yield stability, and quality traits such as cup quality and pest resilience will be critical for translating screenhouse insights into field-ready cultivars. Establishing such integrative trait networks will not only deepen our understanding of drought adaptation in *C. canephora* but also accelerate the development of climate-resilient varieties optimized for sustainable production in water-limited environments.

## Conclusion

This study demonstrates that drought resilience in Ugandan *C. canephora* is driven primarily by genotype‑specific photochemical stability rather than plant vigor or cultivation status. Although drought reduced gas exchange and PSII function across all 165 accessions, a subset of 24 genotypes maintained superior water relations and sustained PSII efficiency under severe stress. Photosynthetic performance index (PI) and Fv/F₀ were the strongest predictors of drought fitness, explaining 55% and 57% of DFI variance, far outperforming gas-exchange traits and pre-dawn water potential (R²adj < 0.05). These results identify photochemical integrity as a core mechanism of drought tolerance in *C. canephora* and highlight PI and Fv/F₀ as operationally powerful selection traits. Incorporating these traits into breeding pipelines will increase selection accuracy and accelerate the development of climate‑resilient coffee cultivars for drought‑prone regions.

## Supporting information

S1 Fig Seasonal temperature and humidity trends inside the screenhouse. Average relative humidity (left axis) and temperature (right axis) recorded from May to December 2024 using seven Tinytag data loggers installed across the screenhouse to have a good spatial distribution of Tinytag sensors, showing pre-stress and drought stress periods.
(TIF)

S2 Fig Phenotypic clustering of *C. canephor*a genotypes based on growth traits. A, Elbow plot showing optimal clustering at k = 2; B, PCA biplot of standardized growth traits, with genotypes grouped into two clusters: cluster 1 (smaller biomass) (blue) and cluster 2 (larger biomass) (red).
(TIFF)

S1 Table Descriptive and comparative statistical analysis of 11 morphophysiological traits in drought stress and well-watered conditions across clusters, over all measurement points. Trt: treatment corresponding to Day 1 (WW) or Day 15 (DS); Min: minimum value of the trait within group (raw), Max: maximum value of the trait within group (raw), Mean ± SE: estimated marginal mean and standard error from the mixed model accounting for batch (emmeans); SD: standard deviation within group (raw); CV (%): coefficient of variation computed from raw values (raw).
(CSV)

S2 Table Mean square ANOVA results from a spline-based linear mixed model analyzing physiological traits. Significance levels are denoted by asterisks: * p < 0.05, *** p < 0.001; ns indicates non-significant results.
(CSV)

S3 Fig Temporal trajectories of key morpho-physiological traits in C. canephora clusters under increasing drought stress. Each panel shows model-derived estimated marginal means (± confidence intervals of the estimated marginal means, reflecting uncertainty of the modeled means rather than the variability among individual plants) from spline-based mixed models across stress days (Day 1 to Day 15) for the small biomass cluster (blue) and big biomass cluster (red).
(TIFF)

S4 Fig Identification of drought response phenotypes using k-means clustering. (Left) Elbow plot depicting the within-cluster sum of squares (WSS) across candidate cluster number (k = 3) indicating the optimal partitioning of the dataset. (b) Distribution of the DFI across the three identified response groups: Tolerant (n = 24), Intermediate (n = 91), and Sensitive (n = 50).
(TIFF)

S5 Fig Pearson correlations among morphophysiological traits in C. canephora on day 15 of drought stress. The heat map displays correlations between the DFI, Y(II), ETR, Fv/Fm, Fv/Fo, Fo/Fm, PI], gs, E, Chlo, PDLWP, and Leafincl. Circle color indicates correlation direction (blue = positive, red = negative), and circle size scales with absolute correlation strength (|r|). Pearson’s r coefficients are displayed within each circle.
(TIFF)

S6 Fig Linear regressions between DFI as independent variable and morphophysiological traits as responsive variables at day 1 (WW conditions). (TIFF)

S7 Fig Linear regressions between DFI as independent variable and morphophysiological traits as responsive variables at day 15 (DS conditions). (TIFF)
