# Correction: Intraspecific drought tolerance in Ugandan *Coffea canephora* for accelerated breeding selection

**DOI:** 10.1371/journal.pone.0352818

**Published:** 2026-06-29

**Authors:** Milton Ali, Settumba B. Mukasa, Valerie Poncet, Godfrey Sseremba, Pierre Marraccini, Pascal Musoli, Daphne Nyachaki Bitalo, Hervé Etienne, Mildred Ochwo Ssemakula, Michael Kanaabi, Doreen Murenju Chelangat, Denis Fabre, Mildred Julian Nakanwagi, Sophie Léran, Anitah Tusiimire, Naome Aryatwijuka, Qurayish Musinguzi, Geofrey Arinaitwe, Boris Delahaie, Mathieu Gonin

The last two authors, Boris Delahaie and Mathieu Gonin, should be noted as contributing equally to this work.
