# Supplementary figures and images for: Intraspecific drought tolerance in Ugandan Coffea canephora for accelerated breeding selection
Source: PLoS One. 2026 May 26;21(5):e0349873. doi: 10.1371/journal.pone.0349873 (PMC13210392; doi:10.1371/journal.pone.0349873)

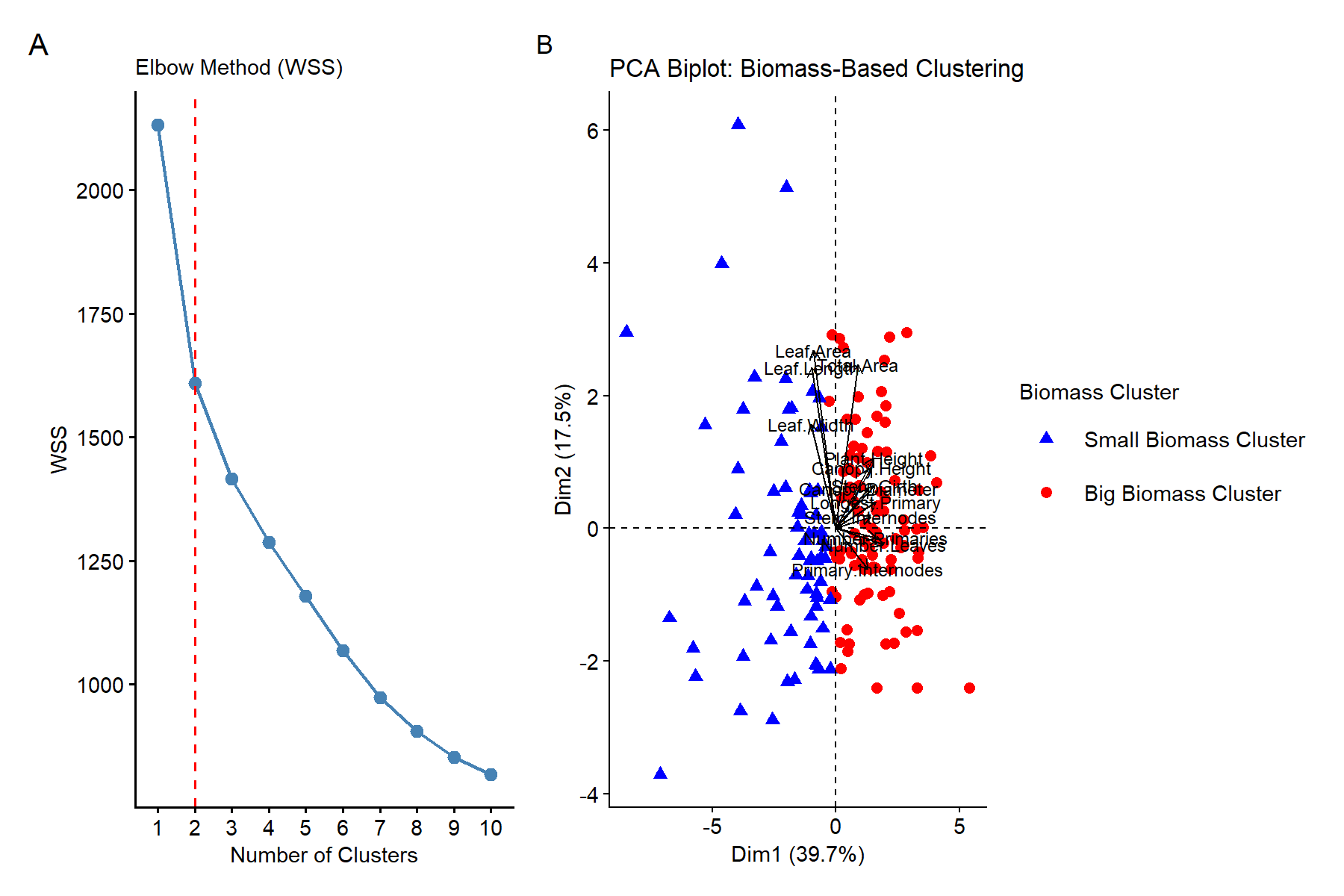

Supplement: S2 Fig — A, Elbow plot showing optimal clustering at k = 2; B, PCA biplot of standardized growth traits, with genotypes grouped into two clusters: cluster 1 (smaller biomass) (blue) and cluster 2 (larger biomass) (red). (TIFF) [file pone.0349873.s002.tiff]

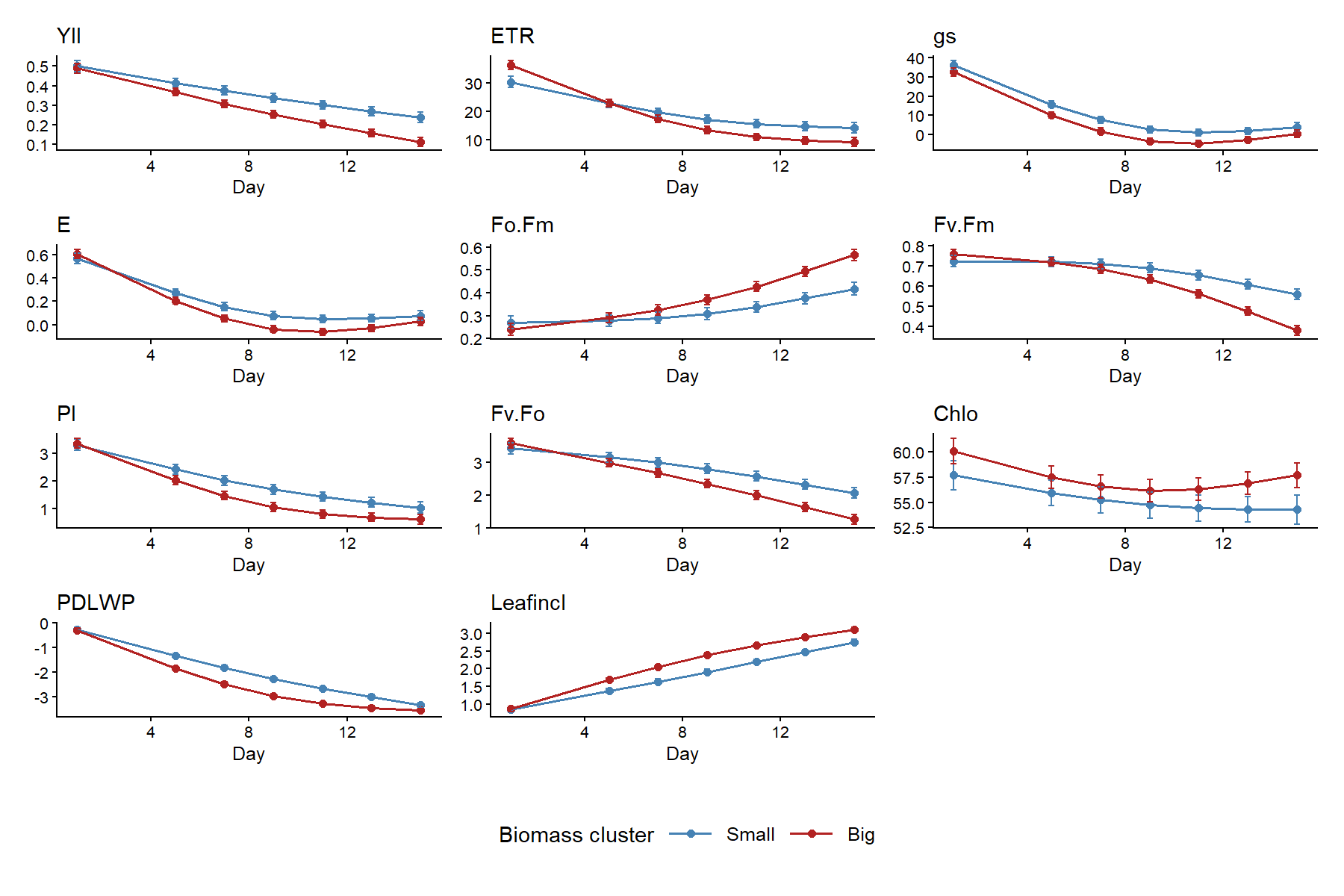

Supplement: S3 Fig — Each panel shows model-derived estimated marginal means (± confidence intervals of the estimated marginal means, reflecting uncertainty of the modeled means rather than the variability among individual plants) from spline-based mixed models across stress days (Day 1 to Day 15) for the small biomass cluster (blue) and big biomass cluster (red). (TIFF) [file pone.0349873.s005.tiff]

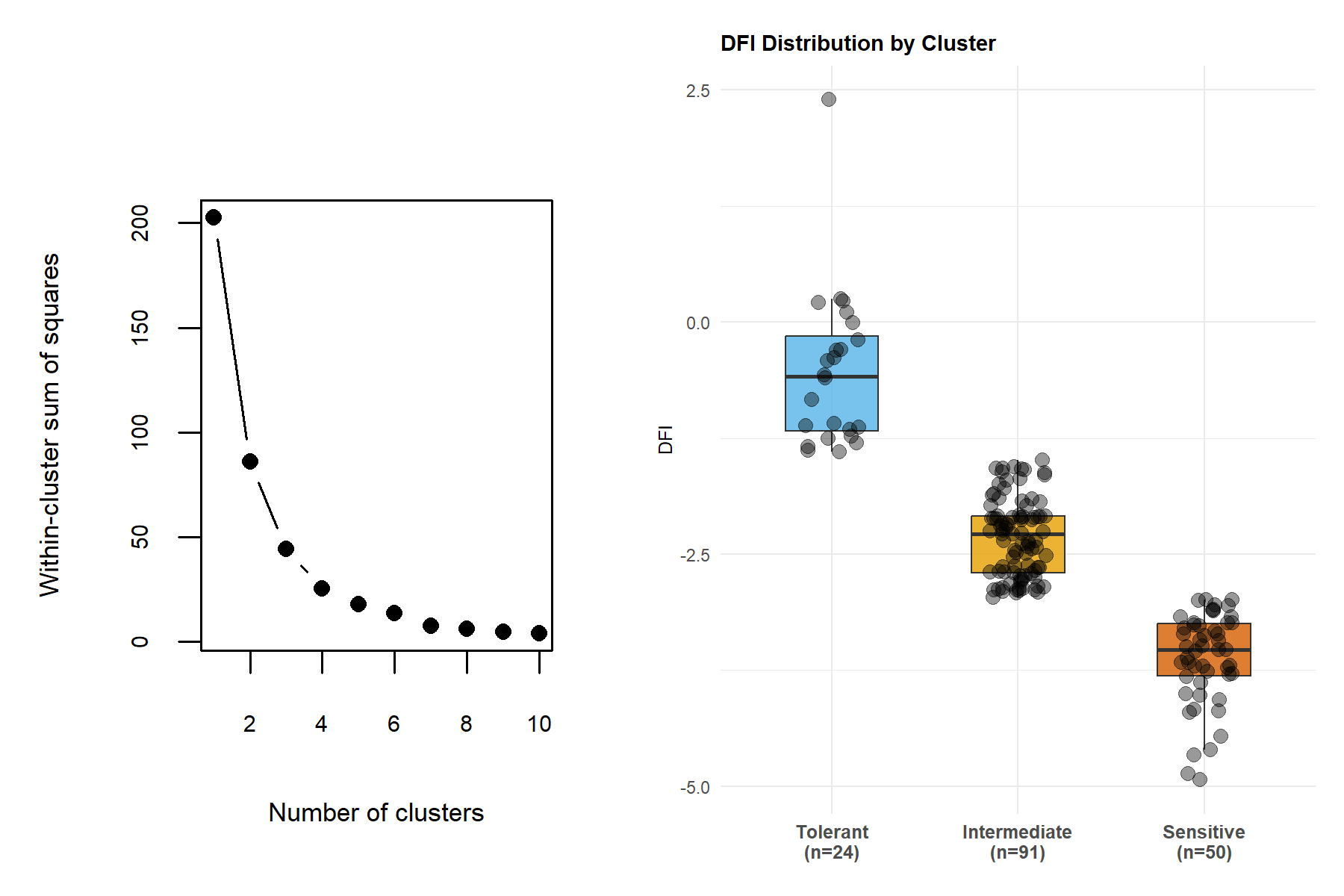

Supplement: S4 Fig — (Left) Elbow plot depicting the within-cluster sum of squares (WSS) across candidate cluster number (k = 3) indicating the optimal partitioning of the dataset. (b) Distribution of the DFI across the three identified response groups: Tolerant (n = 24), Intermediate (n = 91), and Sensitive (n = 50). (TIFF) [file pone.0349873.s006.tiff]

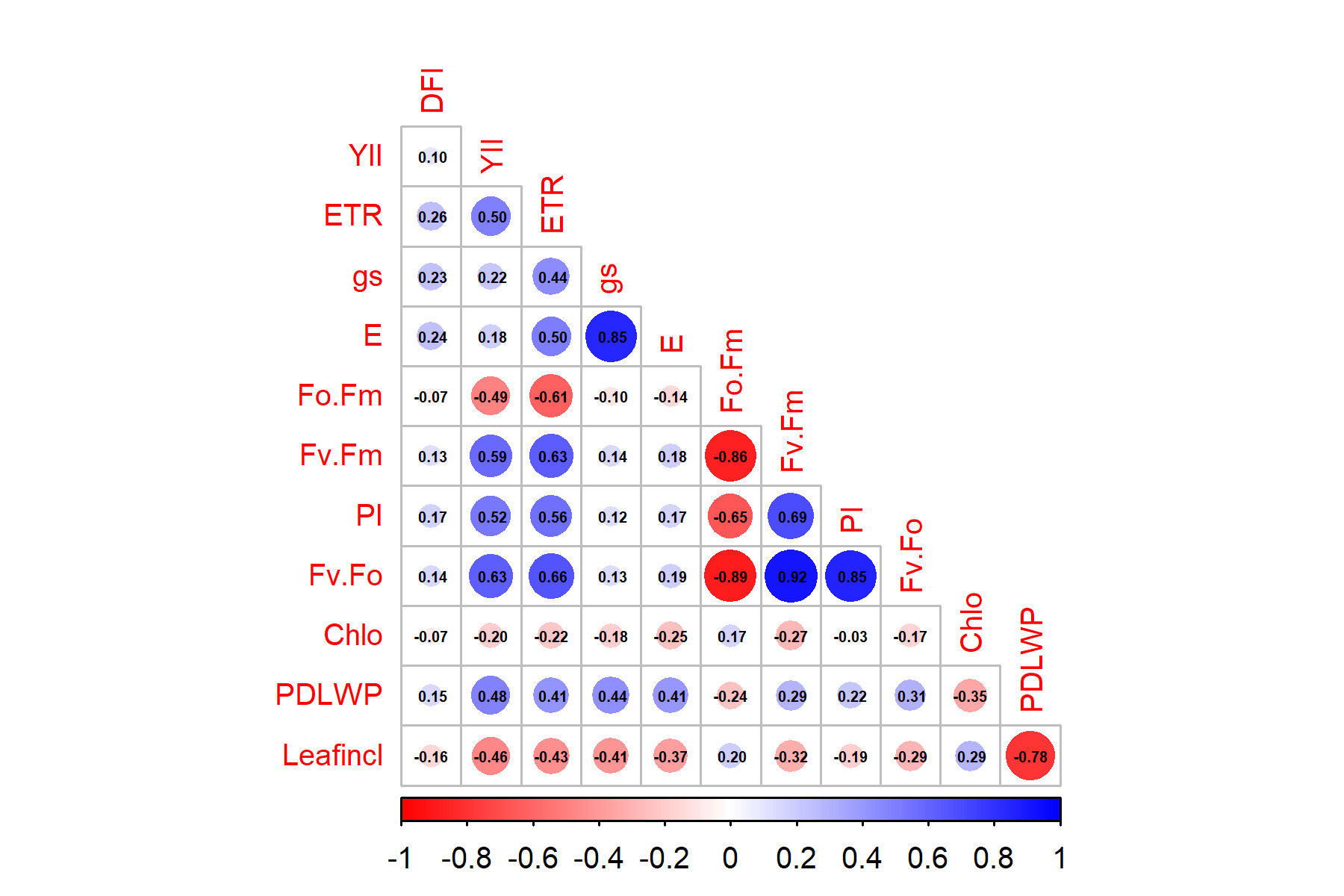

Supplement: S5 Fig — The heat map displays correlations between the DFI, Y(II), ETR, Fv/Fm, Fv/Fo, Fo/Fm, PI], gs, E, Chlo, PDLWP, and Leafincl. Circle color indicates correlation direction (blue = positive, red = negative), and circle size scales with absolute correlation strength (|r|). Pearson’s r coefficients are displayed within each circle. (TIFF) [file pone.0349873.s007.tiff]

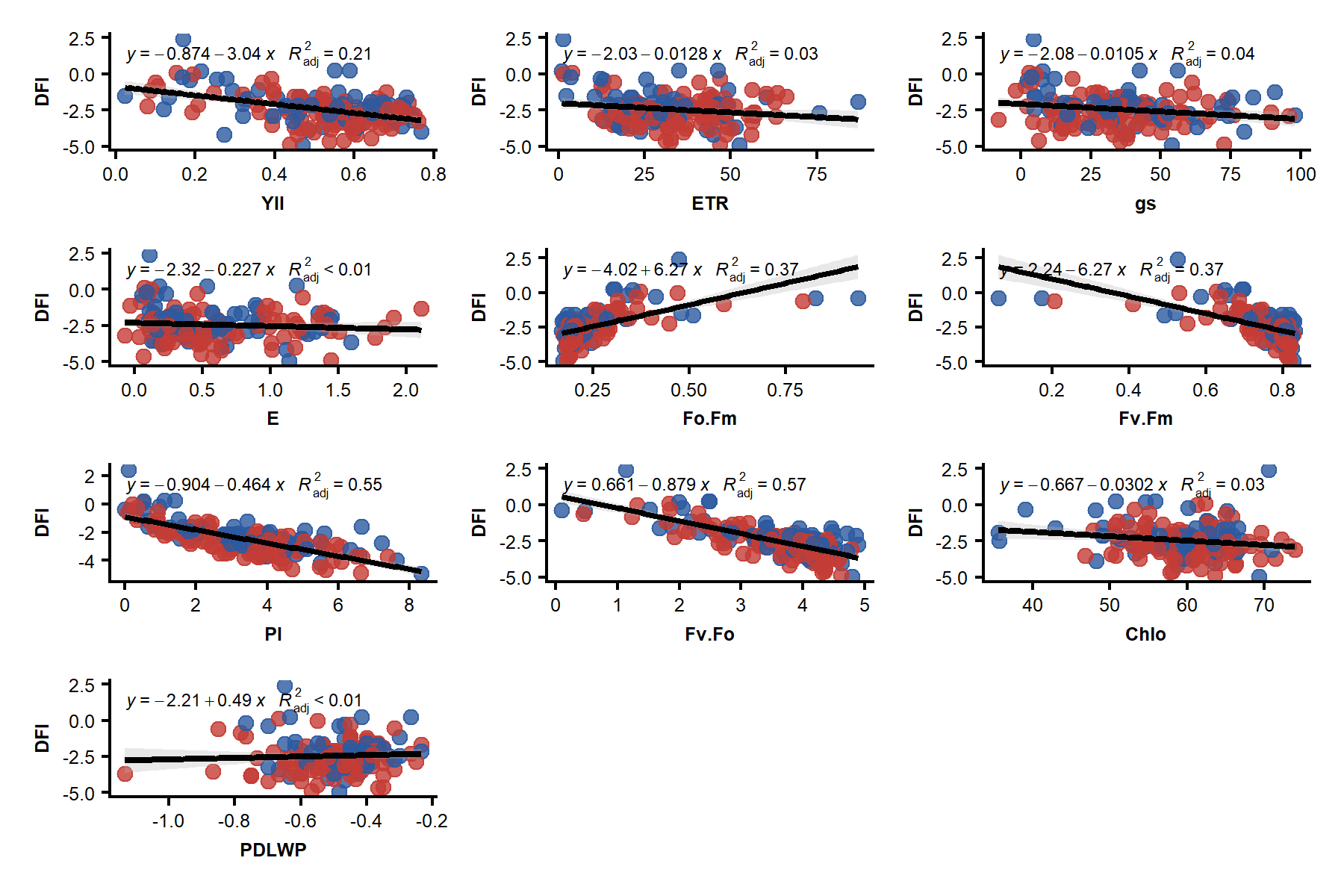

Supplement: S6 Fig — (TIFF) [file pone.0349873.s008.tiff]

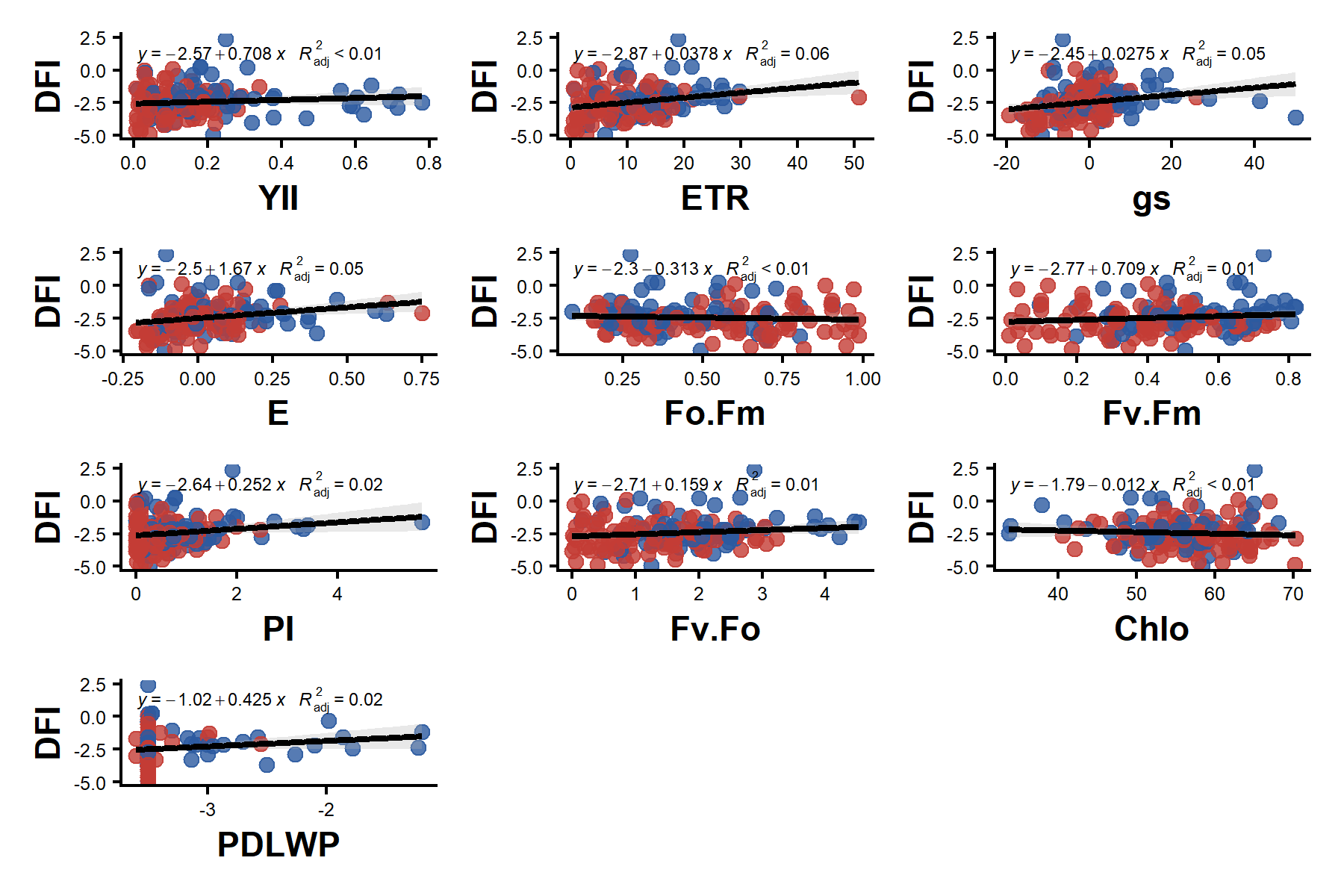

Supplement: S7 Fig — (TIFF) [file pone.0349873.s009.tiff]
